# A127 TIMING OF CHOLECYSTECTOMY AFTER ENDOSCOPIC RETROGRADE CHOLANGIOPANCREATOGRAPHY IN A TERTIARY CENTRE: EVALUATION OF OUTCOMES

**DOI:** 10.1093/jcag/gwac036.127

**Published:** 2023-03-07

**Authors:** C S Liu, D Kao, M Mohamed, D Bigam, S Zepeda-Gomez

**Affiliations:** 1 Department of Medicine; 2 Department of Surgery, University of Alberta Faculty of Medicine and Dentistry, Edmonton, Canada

## Abstract

**Background:**

Endoscopic retrograde cholangiopancreatography (ERCP) is the treatment of choice for patients with choledocholithiasis. Early cholecystectomy (within 24 to 72 hours) is recommended after the initial ERCP to reduce the risk of subsequent biliary events.

**Purpose:**

To investigate the timing of cholecystectomy after ERCP in patients with choledocholithiasis and its associated outcomes in a single tertiary care centre.

**Method:**

This is a retrospective analysis of adult patients who underwent cholecystectomy after ERCP from August 2021 to April 2022 at the University of Alberta Hospital. Outcomes data were stratified according to the length of time between ERCP and cholecystectomy, within 72 hours (early) or after 72 hours (delayed).

**Result(s):**

During the study period, 55 subjects were examined. Indications for ERCP included gallstone pancreatitis (24/55, 44%), choledocholithiasis (19/55, 34%), and acute cholangitis (12/55, 21%).

In total, 30 (55%) subjects received cholecystectomy within 72 hours, while 25 (45%) subjects received cholecystectomy after 72 hours. The two groups were comparable in age, sex ratios, and comorbidities. Out of the patients who received cholecystectomy after 72 hours, 8 (32%) subjects received their cholecystectomy on a subsequent admission. Of these, 2 subjects developed recurrent biliary events before their cholecystectomy, and 1 subject required a conversion to open cholecystectomy. There were no recurrent biliary events amongst the individuals with early cholecystectomy.

Subjects who received early cholecystectomy had a shorter total hospital stay compared to those with delayed cholecystectomy (4.5 days vs 7.3 days, p=0.0002). There was no significant difference between early and late cholecystectomy in conversion rate (3% vs 8%, p=0.58), average operating time (86min vs 83min, p=0.79), intraoperative complications including adhesions (13% vs 12%, p>0.05) and empyema (27% vs 28%, p>0.05), as well as histological rate of chronic cholecystitis (88% vs 92%, p=0.68).

Reasons associated with significantly delayed (>7 days) cholecystectomy after ERCP (n=12) include requiring coordination/consultation with other services prior to operation (3 subjects), prolonged course of gallstone pancreatitis (3 subjects), poor candidate for operation due to comorbidities (2 subjects), surgical cancellation/delays (2 subjects), post-ERCP pancreatitis (1 subject), and patient preference (1 subject).

**Image:**

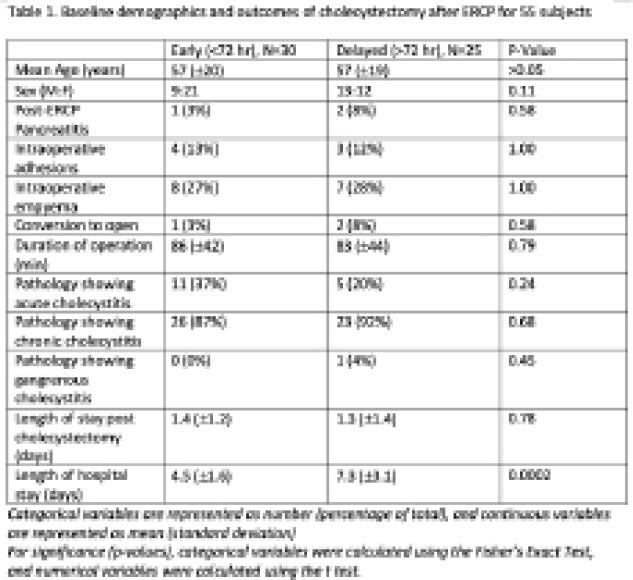

**Conclusion(s):**

Early cholecystectomy is associated with a shorter length of hospital stay and absence of recurrent biliary events. Other post-cholecystectomy outcomes were comparable. Early laparoscopic cholecystectomy should continue to be encouraged through an interdisciplinary approach.

**Please acknowledge all funding agencies by checking the applicable boxes below:**

None

**Disclosure of Interest:**

None Declared

